# A Millifluidic System for Analysis of *Daphnia magna* Locomotory Responses to Water-born Toxicants

**DOI:** 10.1038/s41598-017-17892-z

**Published:** 2017-12-14

**Authors:** Yushi Huang, Olivia Campana, Donald Wlodkowic

**Affiliations:** 1 School of Science, RMIT University, Melbourne, VIC Australia; 2 Instituto de Ciencias Marinas de Andalucia, CSIC, Cadiz, Spain; 3 Centre for Additive Manufacturing, School of Engineering, RMIT University, Melbourne, VIC 3083 Australia

## Abstract

Aquatic toxicity testing in environmental monitoring and chemical risk assessment is critical to assess water quality for human use as well as predict impact of pollutants on ecosystems. In recent years, studies have increasingly focused on the relevance of sub-lethal effects of environmental contaminants. Sub-lethal toxicity endpoints such as behavioural responses are highly integrative and have distinct benefits for assessing water quality because they occur rapidly and thus can be used to sense the presence of toxicants. Our work describes a Lab-on-a-Chip system for the automated analysis of freshwater cladoceran *Daphnia magna* locomotory responses to water-born toxicants. The design combines a Lab-on-a-Chip system for *Daphnia sp*. culture under perfusion with time-resolved videomicroscopy and software tracking locomotory activity of multiple specimens. The application of the system to analyse the swimming behaviour of water fleas exposed to different concentrations of water-born toxicants demonstrated that Lab-on-a-Chip devices can become important research tools for behavioural ecotoxicology and water quality biomonitoring.

## Introduction

Maintaining clean water resources is critical for the integrity of environment and for sustainable supply of water that is safe and suitable for drinking and agricultural use. Contamination of water often occurs as slow cumulative processes. It can, however, also develop quickly following accidental spills or deliberate contamination. It is thus important to develop techniques capable of rapid monitoring of water quality, especially in drinking and agricultural water distribution systems. Although extensive conventional chemical assessments are routinely performed, they are time consuming, expensive, and have an inherently restricted sampling frequency. In addition, chemical analysis does not currently provide continuous analysis with on-line sensing, and as such is incapable of providing early warning and ongoing assessment of suddenly developing toxic hazards.

Apart from chemical analysis, commonly employed acute toxicity biotests are carried out by checking organisms’ survival at pre-defined time intervals (i.e. 24, 48 or 72 h) and estimating median lethal concentrations (LC_50_) of the target chemical or chemical mixtures^1^. Such bioassays based solely on survival endpoints are time-consuming and provide no kinetic information about the toxicological effects of chemicals^2^. They also do not monitor effects that can occur at sub-lethal concentrations of chemicals.

An appealing method that can supplement the above water quality tests is behavioural bioindication. The latter detects environmental changes based on monitoring of behavioural responses of sentinel organisms often referred to as bioindicators^3^. Behavioural responses reportedly precede mortality and are gradually gaining acceptance as sensitive endpoints of acute and sub-lethal stress^3–5^. They can also offer valuable information about the toxic effects of contaminants at environmentally relevant concentrations^5–9^. The latter are usually considerably lower than those detected by conventional acute toxicity tests. For example, it was reported that behavioural endpoints have up to hundred fold higher sensitivity than traditional mortality endpoints^10,11^. Untersteiner *et al*. proved that *D. magna* exposed to 10 µg·L^−1^ copper for 14 h can express significant decrease in swimming velocity while 30 µg·L^−1^ was the 48 h LC_50_ concentration^12^.

Although behavioural endpoints do not provide data about the composition or concentration of toxicants, they provide overall early-warning information about harmful alteration of water parameters^13,14^. Since behavioural parameter often precede physiological, developmental or reproductive effects, they are particularly suited to be used in water biomonitoring technologies^2,4^ employed as early warning systems that in case of sudden water contamination generate an alarm, forming the starting point of a decision and action tree for further analytical and remedial responses^5,14,15^.

The absence of inexpensive and automated instrumentation has so far been a bottleneck in developing the next generation water biomonitoring systems^3,5^. Behavioural tests conducted in multititer plates or large vessels are usually not applicable for studying toxicant avoidance behaviours. This is because of turbulent flow properties that lead to rapid mixing of chemicals. Moreover, analysis of locomotory activities in conventional flasks and vessels is problematic due to limitations of culture vessels that are not optimized for video data recording^5^. The later requires high-resolution optical data acquisition to reconstruct animal trajectories and analyze a multitude of behavioral parameters.

Historically, aquatic ecotoxicity testing has been hampered by the lack of data acquisition automation and analysis algorithms^5^. Even the most basic of readouts that are easily amenable for automated assessment in high-throughput such as e.g. measurements of organism viability or growth with high accuracy, have largely seen no dedicated laboratory automation^5^. In this context progress in automated water biomonitoring systems has been very slow with just a handful of examples developed worldwide. Most recently, Chevalier *et al*. reported a multi-cell static exposure system for continuous tracking of *Daphnia sp*. in which manual recording was no longer needed^16^. Lechelt *et al*. demonstrated a single chamber perfusion DaphTox II system (Bbe moldaenke GmBH) to study surface water pollution, in which multiple behavioural parameters of *D. magna* were adopted to detect presence of toxic substances^17^. Häder and Erzinger have reported on a Daphniatox instrument utilizing static exposure and a computerized image analysis tracking swimming organisms to evaluate up to 14 endpoints including motility, swimming velocity, orientation with respect to light and gravity as well as cell form and size^5^.

The performance of a biomonitoring system based on alterations in fish swimming activity under both laboratory and field conditions was showed by Baldwin and collegues^18,19^. Despite recent progress, most technologies demonstrated to date are large and low-throughput systems hampered by lack of miniaturization and automation. This limits their applicability for real-time water biosensing and high-throughput ecotoxicological analysis^20,21^.

The application of integrated Lab-on-a-Chip (LOC) technologies for automated and long-term culturing of bioindicators can help to overcome the above limitations and lead to a renaissance in water biomonitoring systems^20,22^. Surprisingly, despite substantial potential of LOC in the next generation of environmental monitoring there was so far very little interest in LOC technologies for aquatic ecotoxicology^23–25^. Some recent applications of LOC in aquatic risk assessment include microfluidic systems for automated fish embryo toxicity (FET) biotests as well as toxicity tests using the marine crustaceans^23–25^. No LOC systems were so far developed for freshwater water quality assessment. Such systems have to incorporate not only organism culture, but also electronic tracking and complex on-the-fly computational analysis of behavioural responses exhibited by bioindicators.

To address these issues, we present am integrated millifluidic technology that employs freshwater crustacean *Daphnia magna* as bioindicator species. Changes in *Daphnia sp*. locomotory responses in the presence of toxicants are easy to record optically^12,26,27^. Moreover, their living requirements and long lifespan allow to continuously maintaining them “caged” on LOC devices while sampling water and monitoring behavioural changes, all within a single, automated laboratory system.

This study was aimed at development and validation of proof-of-concept millifluidic chip-based system for aquatic behavioural ecotoxicology and water quality biomonitoring. The main objectives were: (i) validation of the capability to culture freshwater crustacean Daphnia sp. under continuous perfusion; (ii) development of a low cost system to quantitatively assess locomotory traits of sentinel organism using time-resolved videomicroscopy; (iii) development of a chip-based behavioural ecotoxicity test; and (iv) application of the system to analyse perturbation in locomotory behaviour of *D. magna* exposed to sub-lethal concentrations of water-born toxicants. We provide evidence that LOC biomonitoring platform demonstrated consistency of the assessment criteria between toxicity biotests performed on the chip-based device and those recorded by traditional ecotoxicological methods. Moreover, the automated analysis of locomotory activity of *D. magna* exposed to organic and inorganic contaminants provided capabilities not readily achieved using conventional analysis.

## Results and Discussion

### Lab-on-a-Chip technology

**Figure 1:**
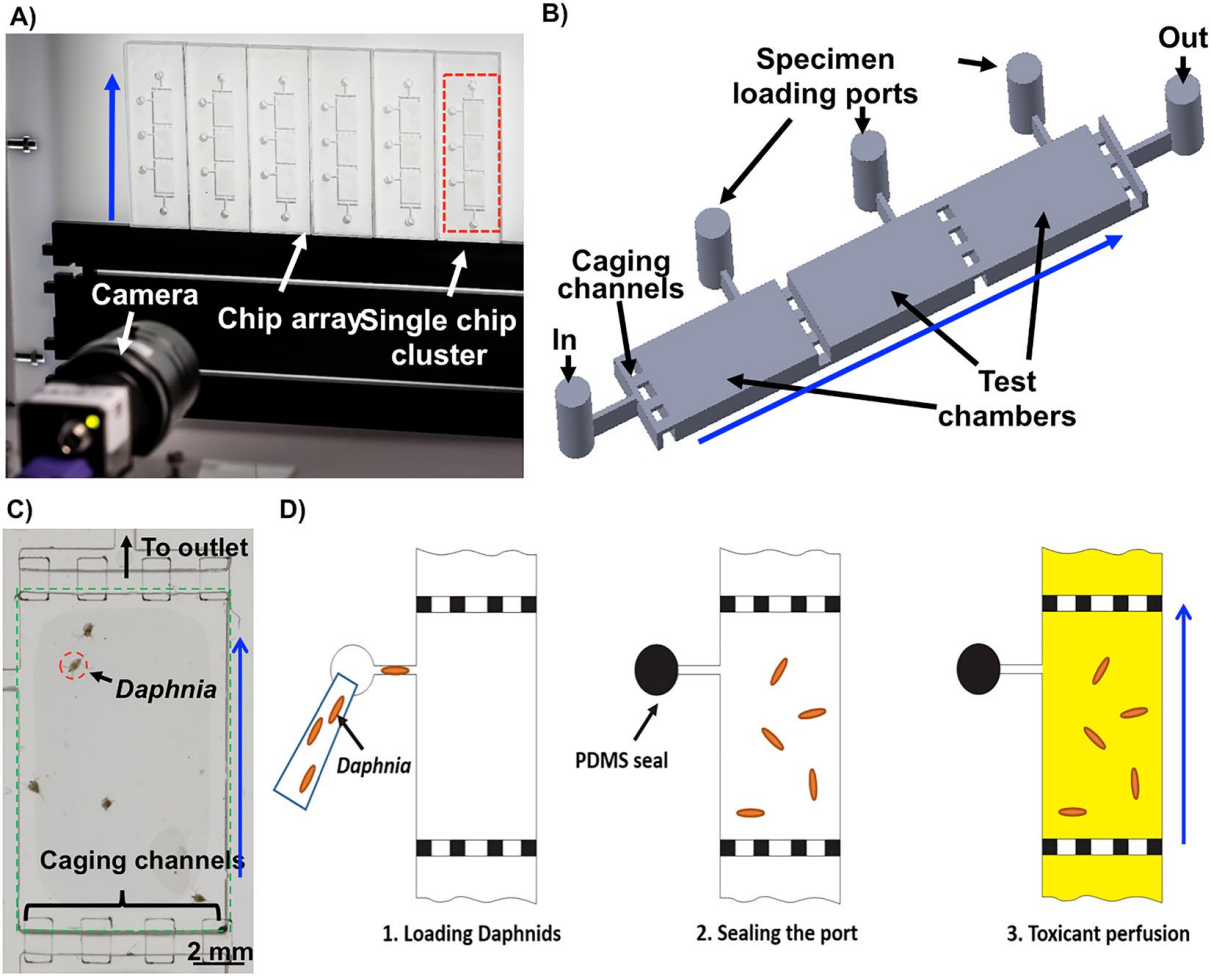
Lab-on-a-Chip system for behavioural toxicity studies on *Daphnia magna* neonates. (**A**) Photograph of the system setup that consists of a millifluidic chip-based array, fluid actuation and optical detection modules; (**B**) 3D computer assisted design (CAD) model of a single millifluidic chip module. It contains chambers that enable caging of *Daphnia* neonates for behavioural toxicity tests; (**C**) Magnified view of a single test chamber with five caged *Daphnia magna* neonates; (**D**) Flowchart depicting operation of the millifluidic device: (1) Daphnia neonates are loaded in each test chamber through loading ports; (2) A PDMS plug is inserted to seal the chamber; (3) Toxicant is pumped through the bottom inlet and flows towards the outlets located in the top part of the device. Blue arrows indicate direction of fluid flow.

The Lab-on-a-Chip biomonitoring platform provided integration of a millifluidic array, high-resolution industrial vision system and dedicated video analysis algorithms for animal tracking (Fig. 1A). It was designed to facilitate on chip biotests on *D. magna*, a freshwater cladoceran extensively used in standard ecotoxicity testing due to its high sensitivity to chemical contaminants, easy handling and culturing^6,28,29^. The chip-based array (Fig. 1A,B) was fabricated in poly(methyl methacrylate) (PMMA) plastic by using laser micromilling. The design included an array of 24 cuboid test chambers (13 × 8 × 2 mm in length (L) width (W) and depth (D) respectively) optimized to keep multiple freely swimming specimens under continuous perfusion (Fig. 1B,C). The chambers in the array were grouped in eight clusters of three to provide entire dose-response analysis with statistical replicates of each test condition (Fig. 1A). Interconnected chambers in a cluster had a shared inlet, outlet, and four groups of “caging” channels (each 1 × 1.5 × 0.3 mm in width, length and depth, respectively) (Fig. 1B). The channels’ geometry provided “caging” of animals that were allowed to freely swim inside the chambers (Fig. 1B,C). To increase the fluid transfer inside the chip device, the caging channels were fabricated in different levels (z) to improve flow across a vertical plane of the chambers. Each chamber had its own loading port to introduce the specimens. The chip device had a total thickness of 7 mm with an inner volume of 826 μL and inner surface area of 1362 mm^2^ for the whole chip. Prior to specimen loading the millifluidic array was coated poly(vinyl alcohol) (PVA, 2%, w/v) to help remove air bubbles during priming^30^. Initially, we tested biocompatibility of the coating by exposing neonates in PVA-coated 24-well plates (PVA, 0.1–2%, w/v) and performing standard 24 and 48-hour immobilization tests according to Daphtoxkit-F (MicroBioTests Inc., Belgium) standard operating protocol. Next we performed similar tests in a millifluidic environment where we compared behaviour of *D. magna* nauplii kept in coated and non-coated PMMA chip-based devices perfused at a volumetric flow rate of 5 mL·h^−1^ (see below). According to our results PVA coating up to 2% w/v had no statistically significant effect on viability or behavioural endpoints of *D. magna*.

### *Daphnia magna* culture under millifluidic environment

**Figure 2:**
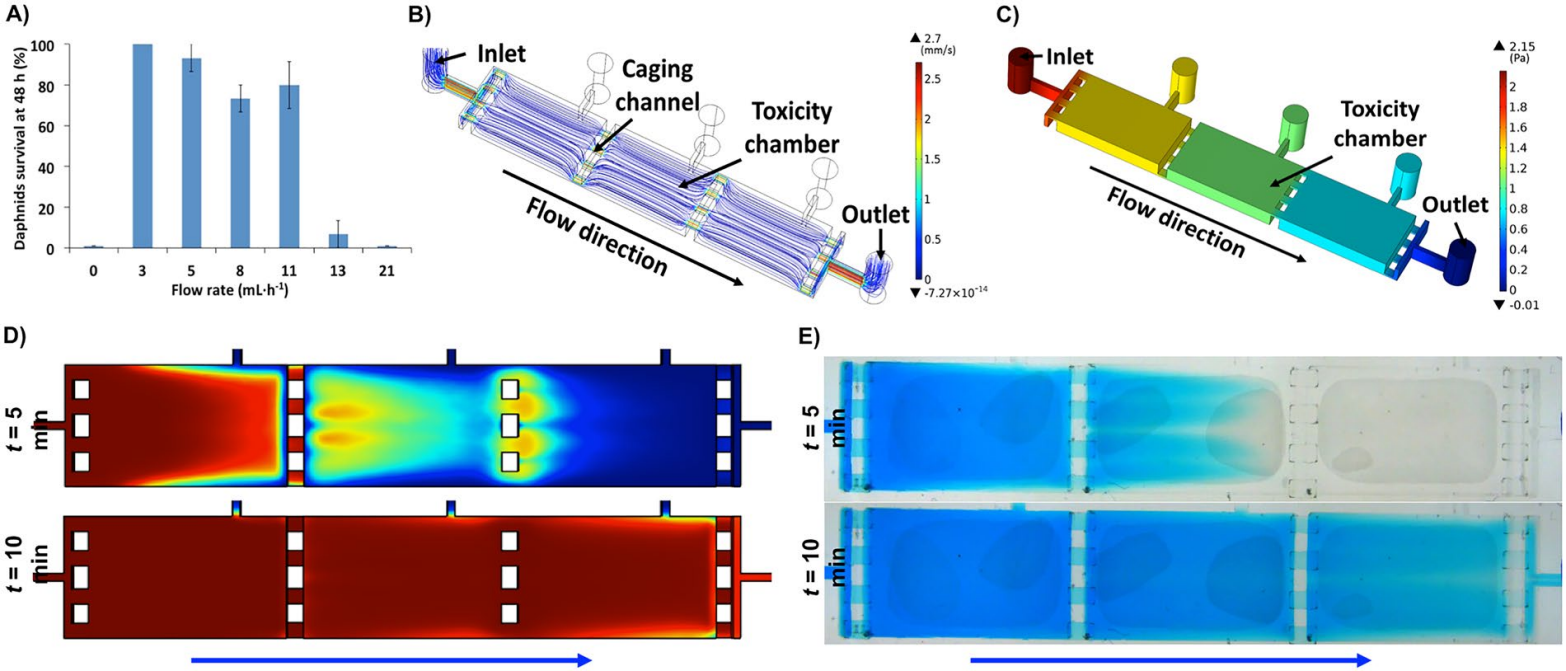
Validation of the Lab-on-a-Chip device for *Daphnia magna* culture under millifluidic environment. (**A**) Survival of neonates under different flow rates (mean ± SE); (**B**) The streamlines of fluid flow across the chip-based device at a flow rate of 5 mL·h^−1^. Streamlines were color-coded by velocity (mm·s^−1^); (**C**) Simulated water pressure (Pa) drop across the Lab-on-a-Chip device at a flow rate of 5 mL·h^−1^; (**D**) Simulated time-resolved mass transfer required for a complete exchange of the dye/toxin inside the caging chambers; (**E**) Experimental validation of the mass transfer efficiency using perfusion with a Trypan blue dye. The time-resolved analysis was performed at a flow rate of 5 mL·h^−1^. Blue arrows indicate direction of fluid flow.

The operating mode of the chip device was a three-step procedure (Fig. 1D). In the first step, the neonates were introduced into the test chamber, then the loading port was then sealed using an elastomeric valve, and lastly the chip perfusion was initiated. Less than 24 h old *Daphnia* neonates hatched from ephipia were used according to a standard operating protocol (Daphtoxkit-F; MicroBioTests Inc., Belgium). Culture conditions were optimised to enable *D. magna* biotests to be adapted to the millifluidic environment. The careful optimization was required because the *Daphnia* neonates exhibit characteristic swimming behaviour that can be easily affected by external factors such as light, temperature, and vibrations. Those microcrustaceans move in characteristic hops using regular beating with the second set of antennae. Movement cycle comprises of alternating hops and brief rest periods. During the resting periods neonates sediment, due to the gravitational force. To enable straightforward monitoring of the hopping behaviour in two-dimensional x-y plane using a video camera, the chip-based devices were designed for vertical mounting configuration (Fig. 1A,C). During biocompatibility experiments five neonates at 24 hours post hatching (hph) stage were randomly selected and loaded into each chamber and the survival rate was recorded based on the number of immobilised neonates after 48 h of continuous perfusion. The locomotory behaviour was analysed as described in detail in the following sections. Native behavioural traits were compared to negative controls comprising of small open chambers of identical size and volume to the millifluidic chips. The main optimization effort was put on attempting to preserve a native movement cycle with alternating “hop and rest” periods as well as sedimentation of immobilized specimens due to the gravitational force. The optimization involved assessing impact of flow rate, flow direction, chamber geometry, temperature and animal density on alterations of locomotion traits. The survival rate of *Daphnia* neonates was monitored under different perfusion rates ranging from 0 to 21 mL·h^−1^. The results indicated over 90 ± 6% (n = 300, ANOVA at p < 0.05) survival rate at flow rates ranging from 3 to 5 mL·h^−1^ (Fig. 2A). These results were in agreement with control culture conditions in multi-well plates where 5–10% mortality (n = 300, ANOVA at p < 0.05) is a commonly observable and accepted value^31^. Under stop flow condition (0 mL·h^−1^), organisms did not survive due to the gas-impermeable PMMA substratum of the chip device that prevented oxygen exchange. At flow rates exceeding 8 mL·h^−1^, neonates were forced against caging channels near the outlets. This led to a significant stress and induced a high degree of mechanical damage that led to a rapid decrease in survival rates (Fig. 2A). It was empirically assessed that at flow rates of between 3 to 5 mL·h^−1^ neonates were able to maintain their normal “hop and rest” swimming pattern without adversary effects of the perfusion on their behavioural activity. Under those conditions the nauplii could be kept healthy for up to 3 days provided that they were fed every day.

Characterisation of the fluidic conditions inside the chip-based device was performed using computational fluid dynamics (CFD) simulations (Fig. 2B,C). The models indicated varying velocities in different domains within the chip device under the simulated perfusion rate of 5 mL·h^−1^. The highest flow velocity of 2.7 mm·s^−1^ was only recorded at the main delivery channels connecting the inlet/outlet with the toxicity chambers (Fig. 2B). The inlet and outlet showed maximum flow velocities of 0.43 to 0.45 mm·s^−1^. Estimated velocities in the toxicity chambers were not exceeding 0.16 mm·s^−1^ (Fig. 2B). Experimental validation of the movement trajectories indicated that such conditions did not affect characteristic patterns of locomotion (data not shown). In those conditions test specimens were exposed to an average pressure drop of 0.2 Pa across each of the chambers (Fig. 2C). This value was lower than shear stress values reported to trigger signalling cascades in isolated cells cultured on chip-based devices^32,33^. Moreover, Zhu *et al*. reported that zebrafish embryos kept on microfluidic device could tolerate a pressure drop of up to 0.6 Pa without any observable impact on zebrafish embryo developmental end-points^24^. Based on the preliminary biocompatibility validation experiments, all toxicity and behavioural tests were carried out at a continuous flow rate of 5 mL·h^−1^ with data acquisition intervals of 1 hour.

Because a homogeneous distribution of toxicants is critical for on-chip toxicity testing^23^, mathematical simulations and physical experiments were performed to characterize mass transfer inside the system. It was estimated that complete exchange of medium can be achieved in approximately 15 minutes at a volumetric flow rate of 5 mL·h^−1^ (Fig. 2D). The simulations were then confirmed by experiments using 0.1% v/v blue food colour dye, showing that 5, 10 and 15 minutes were necessary for a complete exchange of medium in each of the three chambers in a series, respectively (Fig. 2E). The collective data provided a strong correlation between computational models that guided the design of the device and experimental validation showing, that the design will allow for both robust micro-caging of free swimming specimens and uniform mass transfer across the chip-based devices. Experimental results indicated that due to mass transfer limitations the system could not be used for behavioural tests with data acquisition intervals less than 10 min (Fig. 2D,E). However, despite some technical limitations the culture of *Daphnia magna* using millifluidic technology provides some significant bioanalytical advantages. Namely the perfusion provides the ability to temporarily expose the specimens to toxicants. Such exposure can be followed by the rapid exchange with fresh medium. This can be of particular importance for studies on the temporary impact of neurotoxins and/or anaesthetic agents. The latter usually require short-term exposures followed by the recovery in toxin free medium. Such bioassays are notoriously difficult and time consuming to be performed using conventional static conditions. Also analysis of intake of fluorescent-labelled food particles can be greatly facilitated by their quick wash out. The latter can reduce the background fluorescent during subsequent imaging.

### System Integration for Behavioural Analysis

To realize an automated laboratory system for higher throughput behavioural ecotoxicology analysis an integrated interface was developed (Fig. 1A). A custom-made cold light source (light emitting diode (LED) array panel, 5000 K at 500 lux) with an integrated diffuser was used to back illuminate the chip-based array. This allowed for a uniform and shadow-less illumination of all 24 test chambers. The LED panel had an additional functionality as a dedicated holder for the chip-based array. An industrial vision camera equipped with a CMOS sensor characterized by a resolution of 2048 × 2048 pixels (UI-3370CP-C-HQ; IDS Imaging Development Systems GmbH, Germany) and a dedicated lens (focal length 12.5 mm, F1.4-16; Goyo optical Inc., Japan) was integrated as an optical detection system. Most LOC platforms published so far require a motorized microscope stage to achieve data acquisition from multiple samples^34–37^. This can, however, introduce significant challenges in terms of timing coincidence of acquisition events. The latter is particularly problematic for time-resolved video data acquisition from multiple samples. To address this issue, we have created a detection system with no moving stage. Using a chip to detector distance of 25 cm enabled the imaging setup to capture data from up to 24 chambers simultaneously. This provided us with a capability to resolve an entire toxicity dose-response curve in one experiment by using eight clusters with triplicates for each test condition.

**Figure 3:**
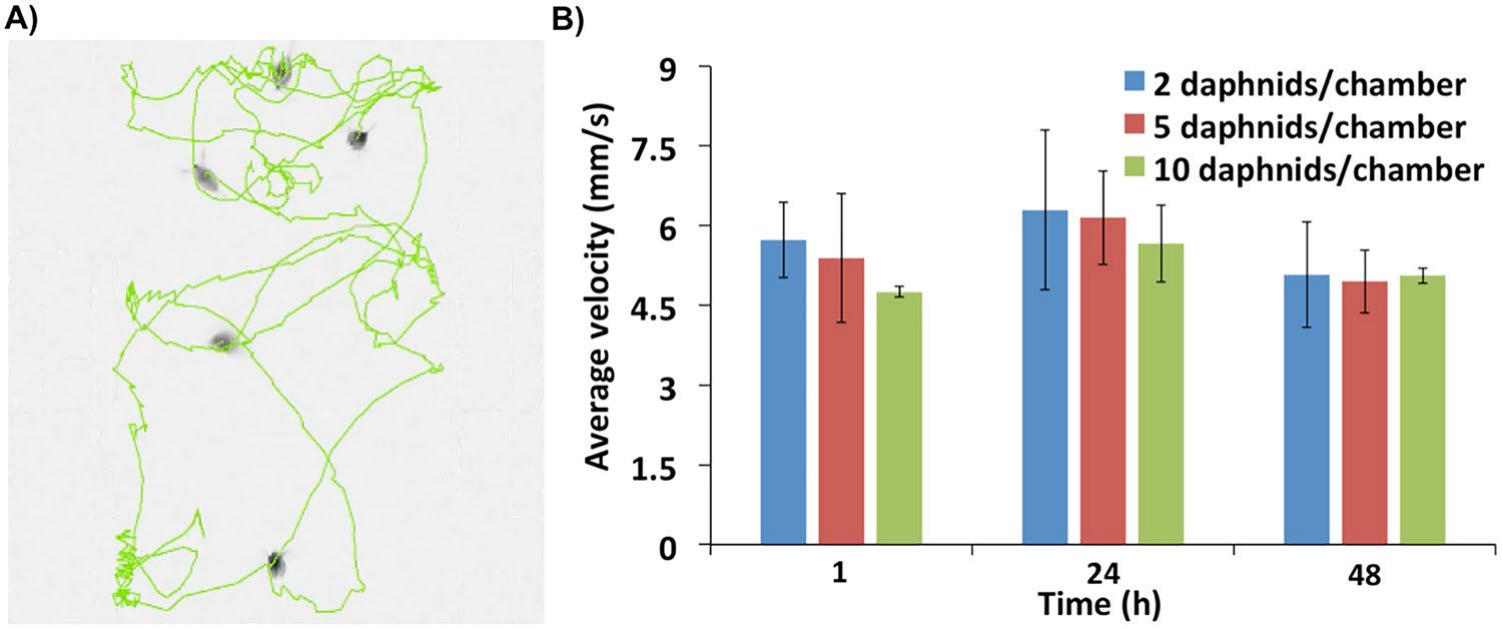
Time-resolved behavioural analysis on millifluidic chip-based devices. (**A**) An exemplary deconvolution of *D. magna’s* trajectories in a caging test chamber; (**B**) Impact of neonate density in the chip device on swimming velocity (mm·s^−1^) (mean ± SE). Data were measured from 15 s long video clips at a frame rate of 30 fps.

The high resolution of the camera sensor provided sufficient optical resolving capabilities to detect 600 μm long neonates kept in each chamber (Fig. 3A). Following acquisition, video clips were deconvoluted for tracking of movement in each frame. Reconstruction of movement trajectories were performed for each individual naupli inside the test chambers (Fig. 3A, Movie S1). The time-stamped coordinates of pixels above chosen threshold were automatically calculated. The data was written into a final file up to thirty times per second. The method avoided any need for manual counting and provided reconstructed specimens’ trajectories as a function of time thus enabling deep mining of behavioural data (i.e. average distance travelled, average speed, acceleration, number of active organisms, etc) for each chip-based device. Due to the limitation in computational power required to process all behavioural data, the system was configured to capture 30-seconds long videos hourly with the rate of 30 frames per second (fps).

The number of objects enclosed in a small area limited the tracing capacity of the analysis software (Fig. 3A). Moreover, the use of many specimens increased the tracking error probability and the time needed to find out the proper settings for tracking algorithms. In order to determine the highest number of organisms that could be tracked with the lowest error probability, we tested the impact of neonates’ density. At the same time the impact of specimen density on their locomotory activity was also evaluated (Fig. 3B). Arbitrary densities of two, five and ten neonates were evaluated under the perfusion of 5 mL·h^−1^, with their behaviour recorded and analysed at 1, 24, and 48 h intervals. Density differences did not significantly affect the average swimming velocity of neonates but the tracking error probability increased when ten neonates were allocated into toxicity chambers (Fig. 3B). Therefore, density test condition was empirically chosen at five neonates per chamber.

### Impact of water-born toxicants on behavioral end-points

As a proof-of-concept of application in aquatic risk assessment, we performed toxicity tests using different classes of chemicals such as (i) heavy metals (copper (II) chloride dehydrate; potassium dichromate); (ii) xanthine alkaloids (caffeine) and (iii) agents with narcotic mode of action (ethanol; dimethyl sulfoxide (DMSO)). All data obtained on chip-based technology were compared to biotests performed according to a standard OECD protocol for acute *D. magna* immobilization test^29^. The aims of application-based validation studies were: (i) to determine if conventional median effect concentrations (EC_50_ at 48 hours) can be generated using LOC device and then can be correlated with the standard OECD ecotoxicity test protocol performed in static multiwell plates; (ii) to assess the behavioural responses of daphnids stimulated with chemicals characterized by different modes of action; and (iii) to assess if time-resolved videomicroscopy analysis can be used to develop sensitive ecotoxicological endpoints.

**Figure 4:**
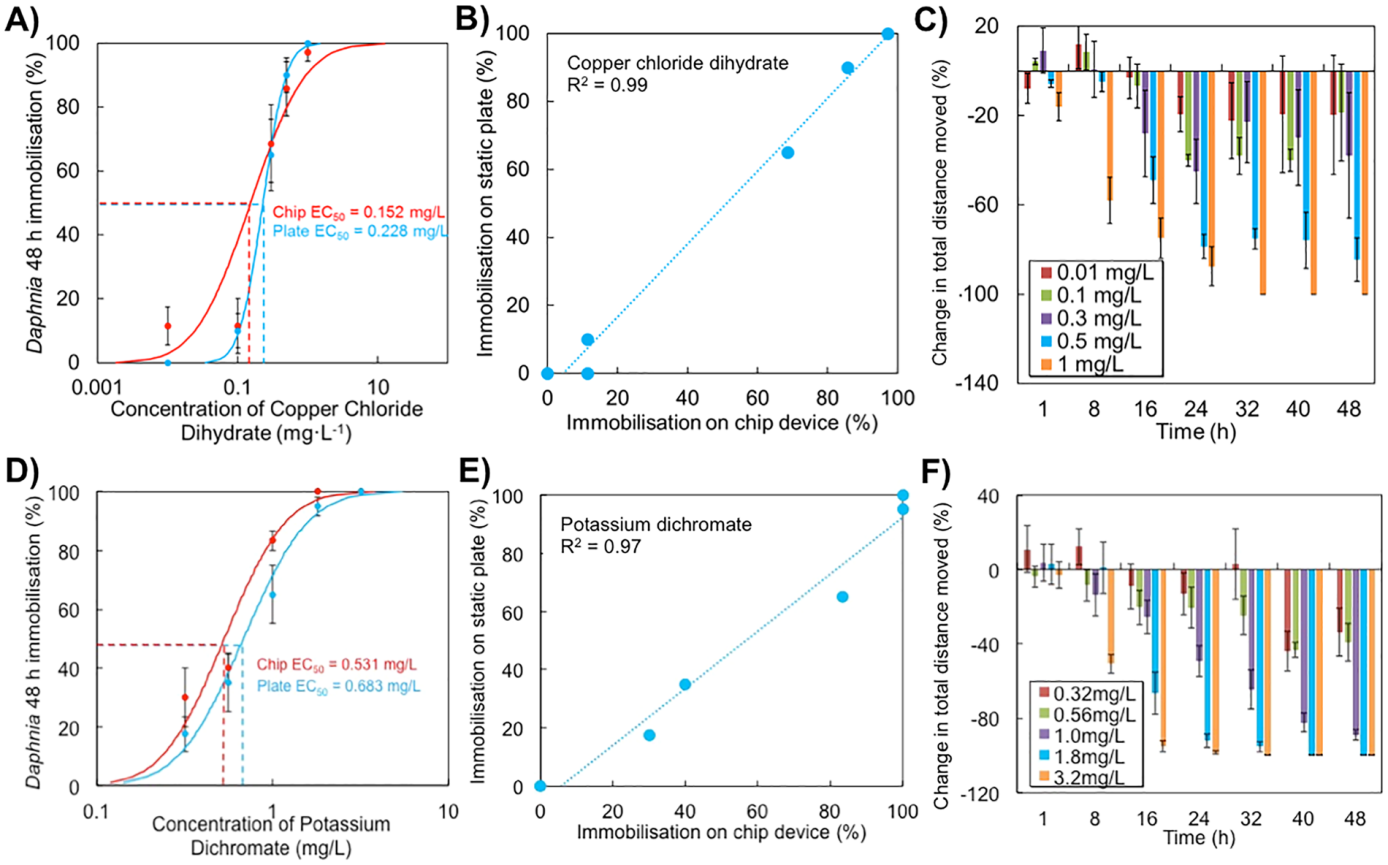
Comparison between conventional and behavioral bioassays on inorganic toxicants performed in a millifluidic environment. (**A**) Standard immobilization tests of *Daphnia magna* neonates exposed to copper chloride. Manual counting was performed according to the standard protocol. Comparative analysis was performed in 24-well microtiter plates under static conditions and Lab-on-a-Chip devices under continuous perfusion. Points represent experimental data (mean ± SE); (**B**) Pearson linear correlation analysis between chip-based and reference conditions; (**C**) Impact of copper chloride on behavioral end-points of *Daphnia* neonates cultured in millifluidic environment. Data was obtained after reconstruction of trajectories. Behavioural responses of neonates, represented as change in total swimming distance (±standard error), exposed to increasing concentrations of copper chloride; (**D**) Standard immobilization tests of *Daphnia magna* neonates exposed to potassium dichromate and obtained using manual counting according to the standard protocol. Comparative analysis was performed as in A). Points represent experimental data (mean ± SE); (**E**) Pearson linear correlation analysis between chip-based and reference conditions; (**F**) Impact of potassium dichromate on behavioral end-points of *Daphnia* neonates cultured in chip-based environment. Data was obtained after reconstruction of animal trajectories. Behavioural responses of neonates, represented as change in total swimming distance (±standard error), exposed to increasing concentrations of potassium dichromate. Positive and negative changes denote hyperactivity and hypoactivity syndromes, respectively. Millifluidic chips were actuated at a constant volumetric flow rate of 5 mL·h^−1^.

Copper is a trace metal widely used in industrial processes and agriculture. As a result, its concentration in the aquatic environment commonly reaches levels toxic to aquatic wildlife^38,39^. The 48 h acute toxicity tests performed on both perfusion-based chip device and standard multi-well plates, resulted in comparable 48 h EC_50_ values of 0.15 and 0.23 mg·L^−1^, respectively (Fig. 4A). Linear correlation analysis performed between results of the two experimental methods had a R^2^ value of 0.99 (Pearson linear correlation test, *p* < 0.01) providing evidence that the millifluidic conditions on a chip did not affect the test outcomes compared to standard toxicity tests (Fig. 4B). The analysis of the behavioural effects showed that copper ions induced a hypolocomotion response on *Daphina* neonates (Fig. 4C). Neonates exposed to 1 mg·L^−1^ copper solution exhibited a significant ~60% decrease in total moving distance after one hour exposure; After 16 h, the neonates exposed to 0.5 mg·L^−1^ copper showed a significant ~50% decrease in their movement (ANOVA; *p* < 0.05). According to experiments conducted by Hansen and Roslev (2016), “distance moved”, expressed as mm/min represented the most relevant endpoint to study the behavioral response of *D. magna* neonates exposed to copper and a binary mixture of glyphosate-Cu^40^. However, they reported a significant inhibition of the distance moved after 48 h exposure at concentrations one order of magnitude higher than those observed in this study^40^. Interestingly, statistically significant 20 to 40% hypolocomotion responses were also observed at concentrations close to EC_10_ values (0.01–0.1 mg·L^−1^) after 24 h of exposure. At this time all specimens remained viable and would not be scored as immobile according to the conventional test protocol. Only the application of the chip-based analysis of this behavioural endpoint allowed for the early detection (1 h time frame) of potential toxic effects at concentration as low as the chronic water quality reference value set at 9 μg Cu L^−1^ by USEPA and considerate protective of freshwater aquatic life. This demonstrates how LOC technology could represent a critical asset when specific effect thresholds of environmental contaminants need to be evaluated.

Potassium dichromate is a common inorganic reagent used in chemical industries as an oxidizing agent. Similarly to all hexavalent chromium compounds K_2_Cr_2_O_7_ exhibits significant acute toxicity to all aquatic life. The 48 h acute toxicity tests performed on both perfusion-based chip device and standard multi-well plates resulted in comparable 48 h EC_50_ values of 0.53 and 0.65 mg·L^−1^, respectively (Fig. 4D). Linear correlation analysis performed between results of the two experimental methods had a R^2^ value of 0.97 (Pearson linear correlation test, *p* < 0.01) (Fig. 4E). During time-resolved behavioural analysis on chip-based devices we observed a significant decrease in locomotory activities of *Daphnia* neonates (20% reduction of average distance covered, (ANOVA; *p* < 0.05) after 16 hours exposure to LC_50_ concentrations. At this time all daphnids remained viable and would not be scored as immobile according to the conventional test protocol. As observed for copper biotest, this data suggest that behavioural analysis can provide more sensitive and faster effect endpoints as compared to the gold standard protocols. The reduction of locomotion was more pronounced with the increase in concentration of the toxicant. Interestingly, exposure to concentrations of potassium dichromate equivalent to EC_10_ values obtained in standard OECD test induced nearly 40% reduction (ANOVA; *p* < 0.05) of average distance covered by the animals still considered to be viable according to a standard manual counting protocol. These concentrations are also similar to those found to inhibit the reproduction activity (EC_50_) in *D. magna* in 21-d toxicity test^34^. This provides further evidence that even delayed behavioral alterations can occur at earlier time points when compared to routinely applied in aquatic ecotoxicology mortality/immobilization endpoints. As such, they can be used as early warning signal of impairment of critical organism life traits that can affect the population dynamics such as growth and reproduction.

**Figure 5:**
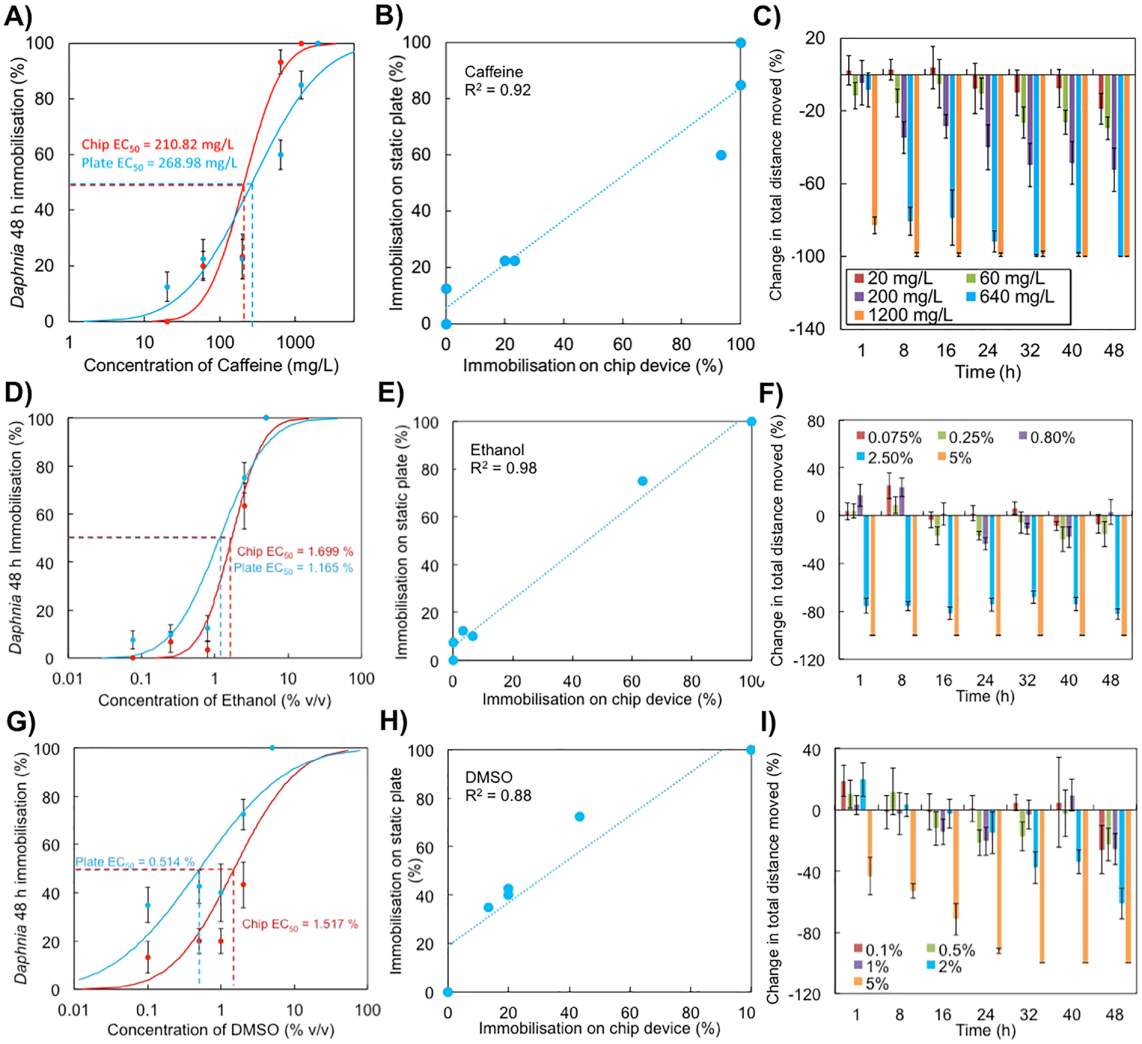
Comparison between conventional and behavioral bioassays on organic toxicants performed in a chip-based device. (**A**) Standard immobilization tests of *Daphnia magna* neonates exposed to caffeine. Analysis was performed in 24-well microtiter plates under static conditions and compared with millifluidic devices. Points represent experimental data (mean ± SE); (**B**) Pearson linear correlation analysis between chip-based and reference conditions; (**C**) Impact of caffeine on behavioral end-points of *Daphnia* neonates cultured in millifluidic environment. Data was obtained after reconstruction of animal trajectories. Behavioural responses, represented as change in total swimming distance ( ± standard error), exposed to increasing concentrations of caffeine; (**D**) Standard immobilization tests of *Daphnia magna* neonates exposed to ethanol and obtained using manual counting according to the standard protocol. Comparative analysis was performed as in A). Points represent experimental data (mean ± SE); (**E**) Pearson linear correlation analysis between chip-based and reference conditions; (**F**) Impact of ethanol on behavioral end-points of *Daphnia* neonates cultured in chip-based devices. Data was obtained after reconstruction of animal trajectories. Behavioural responses of neonates, represented as change in total swimming distance ( ± standard error), exposed to increasing concentrations of ethanol. Positive and negative changes denote hyperactivity and hypoactivity syndromes, respectively. Millifluidic chips were actuated at a constant volumetric flow rate of 5 mL·h^−1^. (**G**,**H**) Comparative analysis of immobilization tests performed in 24-well microtiter plates under static conditions and Lab-on-a-Chip devices under continuous perfusion. *Daphnia magna* neonates were exposed to DMSO and data was obtained using manual counting according to the standard protocol; (**I**) DMSO induced a transient spike of the locomotory response in daphnids when used in concentrations between 0.1 and 2% v/v. The transient hyperactivity was observed immediately after the medium in the test chamber was spiked with the DMSO and lasted for up to 1 hour of exposure.

Caffeine is a xanthine alkaloid consumed largely as beverages and analeptic additives. Over the last decade increasing concentrations of caffeine have been detected in both surface and ground waters and linked to toxic effects on development, reproduction and growth of several aquatic model organisms^41–43^. Tests on *D. magna* indicated that EC_50_ values of 48 h immobilisation tests were 210.8 and 269.0 mg·L^−1^ for the chip device and standard test, respectively (Fig. 5A). Linear correlation analysis performed between the two experimental setups had a R^2^ value of 0.92 (Pearson linear correlation test; *p* < 0.05) (Fig. 5B). Similar to heavy metals, caffeine also induced hypoactivity in *Daphnia* neonates’ (Fig. 5C). At concentrations of 60 and 200 mg·L^−1^ of caffeine that are below EC_50_ values approximately 30 and 50% reduction in locomotory activity (ANOVA; *p* < 0.05) was observed after 8 hours of exposure, respectively (Fig. 5C). At this point animals were still considered to be viable according to a standard manual counting protocol. Subsequent retardation of locomotion increased with the duration of exposure to caffeine. Higher concentrations of caffeine induced dramatic decrease of nauplii movements (80% reduction of total distance covered) after 1 h of exposure to 1200 mg·L^−1^ caffeine (Fig. 5C, ANOVA; *p* < 0.05). A similar result was also observed at a concentration of 640 mg·L^−1^ caffeine already after 8 h of exposure (Fig. 5C, ANOVA; *p* < 0.05).

Neonates of *Daphnia sp*. were also sensitive to chemical exhibiting narcotic mode of action. Experimental LC_50_ values obtained on LOC device correlated well with data collected in a standard static protocol. Accordingly LC_50_ values of 1.7% and 1.2% (v/v) were obtained on-chip vs. reference static conditions (Fig. 5D,E). Time-resolved videomicroscopy revealed that exposure to low concentration of ethanol (0.075–0.8% v/v) induced a transient increase in locomotory activity often referred to as a hyperactivity syndrome. It was defined here as 20% increase in locomotory activity observed between 1 and 8 hours of exposure (Fig. 5F, ANOVA; *p* < 0.05). Those results are in agreement with a recent study by Chevalier *et al*. that reported an ethanol-induced agitation (intense increase of the swimming speed) from the first hour of the ethanol exposure that was subsequently followed by a gradual hypolocomotion syndrome^44^. We have also observed a transient hyperlocomotion followed by a progressive depression of locomotory activity especially at higher concentrations of ethanol. The hyperactivity is often assumed as a rapid attempt to escape the contaminated area. Interestingly, a very stark threshold of responses could behaviorally defined for exposure to ethanol. In this regard concentration above EC_50_ values induced almost complete cessation of locomotors activity defined here as >80% decrease in locomotory activity observed immediately after the medium in the test chamber was exchanged with a solution of the solvent (Fig. 5F, ANOVA; *p* < 0.05). In this regard, the dimethyl sulfoxide (DMSO), widely used for the dissolution of poorly water-soluble hydrophobic chemicals aprotic solvent, also induced a transient spike of the locomotory response in daphnids when used in concentrations between 0.1 and 2% v/v. The transient hyperactivity was observed immediately after the medium in the test chamber was spiked with the DMSO and lasted for up to 1 hour of exposure (Fig. 5I).

The results indicated that by applying integrated LOC technology and automated videomicroscopy for assessing behavioural ecotoxicity endpoints a more sensitive testing could be potentially achieved with higher automation than the conventional *Daphnia* acute toxicity test^29^. Compared to a handful of existing biomonitoring as well as behavioural analysis technologies developed for *Daphnia sp*.^12,15,26,27^ the LOC enabled approach presented here shows a significant potential for a cost-effective, inherently customisable for both research and routine screening applications and enabling higher throughput behavioural bioassays that are not readily achieved using conventional solutions. The currently available systems such as DaphTox II can assess multiple behavioral parameters and keep *Daphnia* cultures fed for over 7 days at a time^17^. Those systems are, however, profoundly limited by the throughput of analysis where for instance DaphTox II can only analyze one sample and one control “cell” each containing up to ten daphnids at a time^17^. Moreover the very low volume of media used by the microfluidic chip-based technology as well as capability to pulse the specimens with toxicants followed by the fresh medium exchange opens up new analytical avenues for neurobehavioral toxicology.

Another interesting point worth discussing is ability of the millifluidic system to perform more realistic analysis of swimming trajectories. Swimming trajectory can be generically defined as a pathway left by a moving organism^14^. It is usually expressed as length measured in millimeters. It can also be defined as a unique shape/pattern. Both parameters can be analyzed in both 2- or 3-dimensions often simply referred to as 2D and 3D, respectively^14,45,46^. Conventional 2D analysis of swimming daphnids is usually performed in open dishes with very low level of water^47^. This forces cladocerans to swim horizontally and introduces a strong analytical bias because such conditions do not reflect natural swimming of daphnia^14,47^. Moreover such experimental conditions preclude observations of traits such as gravitaxis defined here as an orientation and movement with respect of gravitational field of the Earth. In contrast to most conventional 2D methodologies the presented system is oriented vertically and thus enables studies of gravitaxis, change in upward/downward swimming direction as well as downward/upward angular alteration^14^. Moreover because the optical axis is horizontal to the analytical chambers, non-motile and/or dead specimens sediment at the bottom of the chamber and thus the percentage of both motile and non-motile neonates can be rapidly determined^14^.

The main limitation of the presented technology is undoubtedly the inability to perform three-dimensional analysis of trajectories. 3D tracking of specimens provides the most detailed information of natural swimming of *Daphnia sp*.^14,45,46^. However, only a handful of authors have applied 3D parameters in aquatic ecotoxicology studies with daphnids^45,46^. As most studies utilizing 3D tracking were predominantly ecological studies the actual relevance of 3D trajectories and sensitivity enhancement using such tracking in water quality biomonitoring applications remains unclear^14^. Furthermore despite some advantages of 3D tracking systems their significant technical downside is low throughput of analysis and considerably higher costs per sample. It is physically impossible to create dense arrays of chambers that can be simultaneously analyzed in 3D. Moreover there is still no off-the-shelf and user-friendly video analysis software that can rapidly perform 3D analysis and trajectory deconvolution. The latter still remains a tedious semi-automated process that with the progress of cognitive machine learning will be hopefully addressed in the near future.

Furthermore the presented technology is significantly cheaper and easier to operate than any of the commercially available systems. Our custom technology built in house as a proof-of-concept prototype costs approximately 5000 US$ while commercial systems often cost in excess of 100.000 US$. Miniaturized system also enables higher throughput analysis where the entire dose-response curve can be analyzed and resolved in one experiment. Lastly while continuous monitoring of behavioral responses at minute intervals for many hours or days is often deemed advantageous we found that due to inherent limitations of both mass transfer and shear stress in any perfusion-based system the real sampling interval is usually limited to 5–10 minutes. Despite those limitations behavioral analysis exploiting time-resolved videomicroscopy can lead to development of new ecotoxicity end-points that can be more sensitive when compared to protocols that utilize mortality as the main marker. More work is undoubtedly required to comprehensively evaluate correlation between behavioral and other commonly used sub-lethal toxicity endpoints such as development and reproduction. This is particularly valuable for assessment of organic and inorganic pollutants that usually occur in concentrations below experimentally generated EC_10_ values.

## Conclusions

Aquatic toxicity biotests performed on small sentinel organisms are commonly employed in water quality assessment to meet country-specific regulatory standards. Limitations of conventional biotests in terms of their sensitivity thresholds, environmental relevance and rapid readout capability have fueled the increasing interest in alternative sub-lethal bioassays such the behavioral endpoints. The latter when combined with appropriate sensing technologies can provide on-line sensing of water quality and thus provide early warning and ongoing assessment of suddenly developing toxic hazards. Advantages of behavioral endpoints in aquatic ecotoxicology are currently hampered by the lack of user-friendly laboratory automation. The very low-throughput of ecotoxicity biotests is in stark contrast to commonly accepted automation routines in drug discovery.

Our present work provides a prototype of a miniaturized and automated millifluidic system for the enhancement of behavioral aquatic toxicity studies based on tracking of locomotory alterations of small aquatic invertebrates. It enabled development of an ecotoxicity bioassay that dynamically quantifies locomotion activity of *D. magna* kept under perfusion. The combination of video-microscopy and Lab-on-a-Chip platform was successfuly validated with model toxicants. We envisage that behavioural bioassays enabled by LOC systems can provide new analytical tools for aquatic ecotoxicology. Furthermore LOC systems can prospectively be used to develop futuristic water biomonitoring systems for real-time synoptic water quality assessment, applicable for a range of industrial applications.

## Materials and Methods

### Test organisms and treatment

Dormant eggs (ephippia) of the freshwater crustacean *Daphnia magna* were hatched according a standard protocol (MicroBioTests Inc., Belgium). Briefly, ephippia were placed in a Petri dish containing 50 mL of pre-aerated Standard Freshwater. They were then incubated at 21 ± 1 °C with a constant illumination of 7000 lux for up to 80 hours. After hatching animals were fed with a suspension of algae powder for 2 hours. In standard toxicity experiments neonates were exposed to selected concentrations of toxicants for 48 h in 24-well plates. Animals were exposed in groups of 5 per well and dead animals were excluded from the well. The number of immobile organisms were counted manually every 24 h. Organisms were considered immobile in absence of reaction for 10 seconds after gentle prodding. Maximum accepted mortality in negative controls did not exceed 5%. For chip-based experiments five daphnids were randomly selected and loaded into microfluidic chambers. A high-precision, low pulsation peristaltic pump (Miniplus Evolution, Gilson Inc., Middleton, Wisconsin, USA) was then used for perfusion of toxicants.

All toxicants were dissolved in freshwater or DMSO. Where carrier solvent DMSO was used controls always included (i) blind control (pure water) and (ii) vehicle control (carrier solvent only). Toxicity testing protocols as well as statistical analysis chosen adhered to international “OECD Guidelines for the Testing of Chemicals” (http://www.oecd.org/chemicalsafety/testing/oecdguidelinesforthetestingofchemicals.htm).

### Computational Fluid Dynamics (CFD) simulations

Models of millifluidic devices were made in three dimensions using SolidWorks 2013 (Dassault Systems SolidWorks Corp, Concord, MA, USA). The 3D models were then converted to standard .iges files and imported into COMSOL Multiphysics® version 4.4 (COMSOL INC, Burlington, MA, USA) software. The differential equations governing the balance of mass, momentum and chemical species determining the flow characteristics were solved using finite-volume algorithms as described before.

### Fabrication process

Individual layers of the chip-based devices were cut using a non-contact 30 W infrared laser machining system with a 50 μm elliptical laser beam spot (Universal Laser Systems, Scottsdale, AZ, USA). The layers were fabricated in a biocompatible and transparent thermoplastic poly(methyl methacrylate) (PMMA). After cutting PMMA layers were cleaned and manually aligned using optical guide marks. The layers were then thermally bonded in a fan assisted oven for up to 90 minutes at a temperature of 120 °C.

### Videomicroscopy

An UI-3370CP-C-HQ industrial vision camera (IDS Imaging Development Systems GmbH, Germany) was integrated into the system and used for all videomicroscopy experiments. The camera was equipped with a 2 K CMOS sensor with a resolution of 2048 × 2048 pixels. It was paired with a dedicated CS-mount lens with a focal length of 12.5 mm (Goyo optical Inc., Japan). The software enabled programming of time-resolved data acquisition as well as a direct control of both video capture and video exposure parameters. The camera software was calibrated for accurate measurements of distances in x-y space. In all experiments 30 seconds long videos at a rate of 30 frames per seconds (fps) were captured every hour for up to 24 hours of the biotest duration. An array of light emitting diodes (LED, 5000 K at 500 lux) with an integrated diffuser provided a uniform illumination. Application of LEDs eliminated heating up of medium that could otherwise influence behavior of *Daphnia sp*. neonates.

### Behavioural data analysis

Analysis of movements of *Daphnia sp*. was performed using LoliTrack V.4 (Loligo^®^ Systems, Denmark) software. To enable simultaneous analysis of large numbers of specimens multiple instances of the software were run in parallel on a workstation computer designed for very intensive parallel processing tasks. The system employed utilized dual six-core Intel Xeon processors with hyper-threading technology providing 24 virtual computing cores.

The time stamped data depicting the x-y positions of daphnids were automatically extracted from the video files. They were used to plot trajectories and calculate behavioral parameters such as: average distance travelled, average speed, number of active organisms) calculated for each sample. The generated data was specific to each neonate and subsequently averaged for the whole population kept in each chamber. Population-level data for each group were then integrated into a time sequence saved in an Excel file. Normalized data was presented as a % change to control experiments. Independent controls were run for every single experiment and were characterized by less than 5% difference between acquired samples.

### Statistical analysis

Data analysis was performed using Prism 6 (GraphPad Software Inc, La Jolla, CA, USA) and ToxRat Professional (ToxRat Solutions GmbH, Alsdorf, Germany) software packages. Standard dose-response curves were modeled using the Hill model in the Prism 6 and ToxRat Pro. An ANOVA model was applied for behavioral analysis with a Student’s t-test to perform independent comparisons of each toxicant concentration. Data for each concentration and time point was compared to the independently run control tests. Data were presented as percentage change normalised to controls.

### Data availability

The datasets generated during and/or analysed during the current study are available from the corresponding author on reasonable request.

## Electronic supplementary material

Supplementary Movie 1
